# Trace fossil evidence of coral-inhabiting crabs (Cryptochiridae) and its implications for growth and paleobiogeography

**DOI:** 10.1038/srep23443

**Published:** 2016-03-24

**Authors:** Adiël A. Klompmaker, Roger W. Portell, Sancia E.T. van der Meij

**Affiliations:** 1 Florida Museum of Natural History, University of Florida, 1659 Museum Road, PO Box 117800, Gainesville, Florida 32611, USA; 2 Department of Integrative Biology and Museum of Paleontology, University of California, Berkeley, 1005 Valley Life Sciences Building #3140, Berkeley, California 94720, USA; 3 Naturalis Biodiversity Center, Darwinweg 2, 2333 CR Leiden, The Netherlands; 4 Oxford University Museum of Natural History, Parks Road, Oxford OX1 3PW, United Kingdom

## Abstract

Members of the Cryptochiridae are small, fragile, symbiotic crabs that live in domiciles in modern corals. Despite their worldwide occurrence with over 50 species known today, their fossil record is unknown. We provide the first unambiguous evidence of cryptochirids in the fossil record through their crescentic pits, typical for certain cryptochirids, in Western Atlantic fossil corals, while the Eocene genus *Montemagrechirus* is excluded from the Cryptochiridae and referred to Montemagrechiridae fam. nov. Nine Pleistocene corals with crescentic pits originate from Florida (USA), and single specimens with pits come from the late Pleistocene of Cuba and the late Pliocene of Florida, all of which are measured for growth analyses. These pits represent trace fossils named *Galacticus duerri* igen. nov., isp. nov. A study of modern cryptochirid domicile shape (crescentic pit, circular-oval pit, or a true gall) shows that species within crab genera tend to inhabit the same pit shape. Crescentic pits in corals occur not only in the Western Atlantic today, but also in the Indo-West Pacific and in the Eastern Pacific. Thus, examination of Cenozoic fossil coral collections from these regions should yield further examples of cryptochirid pits, which would help to constrain the antiquity of this cryptic crab family.

The majority of modern brachyuran decapod crustacean families have a fossil record with at least one species represented (70/95, 74% 1). However, 25 families are not represented by a species found also or exclusively in the fossil record. One of these families is the Cryptochiridae, although an Eocene species was claimed recently^2^, but its identity was questioned^3^ (see discussion for a further evaluation). These crabs are small (<10 mm) and inhabit modern scleractinian corals by modifying the skeletal structure of the coral so that a pit or, less frequently, a calcareous gall is formed that serves as their domicile. They have been considered parasitic to their coral host^4^, but others^5^,^6^,^7^ did not favor this interpretation (see also a more detailed discussion^3^). The Cryptochiridae currently consists of 52 species arranged in 21 genera^8^,^9^,^10^,^11^,^12^,^13^,^14^,^15^,^16^. All species are obligatory associates of corals^17^. They generally live in tropical, shallow waters, although some species are known to live in deeper waters up to 512 m^18^,^19^,^20^,^21^. Their distribution is necessarily limited by the presence of corals and their known distribution so far is restricted to the (sub)tropical regions of the world. Their highest reported diversity is from the Indo-West Pacific with other occurrences in the Atlantic, Pacific, and Indian Oceans^22^. These crustaceans may serve as a prime example of (1) the relatively small size of crabs in reef environments^23^,^24^, (2) the high diversity of crabs in reefs^23^,^25^,^26^,^27^,^28^,^29^, and (3) the cryptic nature of various crabs in reefs^30^,^31^.

The suprafamilial phylogenetic relationships of the Cryptochiridae within the Brachyura are not yet resolved. Based on sequence data, placement was suggested in the Grapsoidea^22^, but others maintained placement within its own superfamily, the Cryptochiroidea^1^,^32^. Recent phylogenetic work has not provided unequivocal results either. An article from 2014^33^ showed a clade consisting of single species of Xenograpsidae and Cryptochiridae, albeit without support, whereas another article from the same year^34^ argued that cryptochirids are not highly specialized grapsids, and called for more research to elucidate the placement of cryptochirids within the Thoracotremata.

Since only two species (*Hapalocarcinus marsupialis* and *Pseudohapalocarcinus ransoni*) are known to produce true galls, typically made by coral surrounding the crab, the informal name “gall crabs” is confusing because all other cryptochirid species form pits rather than galls (Fig. 1). Moreover, males are thought to be mostly free-living and migrate from coral to coral in search for females^9^,^22^,^35^. In terms of size, males are usually smaller than females^22^,^36^. Cryptochirid carapace length is <10 mm^18^ (maximum carapace length Atlantic cryptochirid males: 4.2 mm, females: 5.8 mm^18^), and the diameter of their pits is of slightly larger size.

These pits have rarely been the focus of biologists, who mostly described the morphology of the cryptochirid skeleton, although an exception exists^37^. As a result, the pit shape is not known for all cryptochirids. The morphologies of cryptochirid domiciles are crescentic or circular to oval pits in addition to true gall-shaped forms.

Their absence in the fossil record^1^,^32^,^38^ is puzzling given their modern diversity, but can be explained by their small size, cryptic nature, and fragile body. All that may be left after the fossilization process is the pits formed as suggested previously^32^,^39^,^40^, but the wide circumtropical distribution could indicate an ancient origin^32^.

Here we provide the first evidence of crescentic pits, which closely match those produced by modern cryptochirids, in late Cenozoic corals from the Western Atlantic (Florida and Cuba). Other goals of this paper are to describe and quantitatively assess these pits, erect a new ichnogenus and ichnospecies, and to outline where more fossil occurrences are to be expected given the modern biogeographic patterns of cryptochirid domiciles. Methods including nomenclatural acts are detailed in Supplementary Discussion S1.

## Results

### Coral specimens with crescentic pits

**Figure 2:**
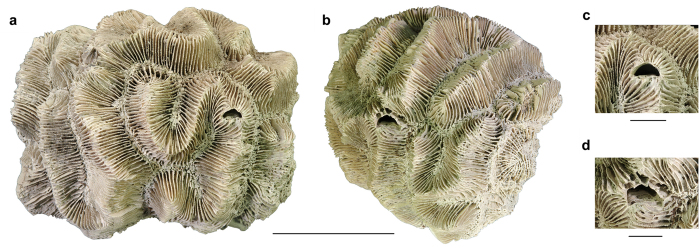
*Manicina areolata* (UF 257523) with two crescentic pits (UF 257524) from the middle Pleistocene Bermont Formation at Palm Beach Aggregates 03, Palm Beach County, Florida. (**a**,**b**) Views of entire coral. (**c**,**d**) Close-ups of both crescentic pits. Scale bar width = 50 mm for (**a**,**b**); 10 mm for (**c**,**d**).

**Figure 3:**
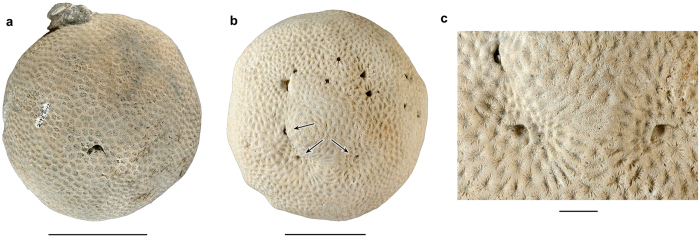
Specimens of *Siderastrea* spp. with crescentic pits. (**a**) Complete coral (UF 242842) with a pit (UF 257626) in *Siderastrea siderea* from the middle Pleistocene Bermont Formation at the GKK Pit 01, Palm Beach County, Florida. (**b**,**c**) Complete coral (UF 24954) and close-up of three pits (UF 257628) in *Siderastrea* cf. *dalli* from the lower Pleistocene Caloosahatchee Formation at the Cochran Shell Pit, Hendry County, Florida. Scale bar width = 50 mm for (**a**,**b**); 10 mm for (**c**).

**Figure 4:**
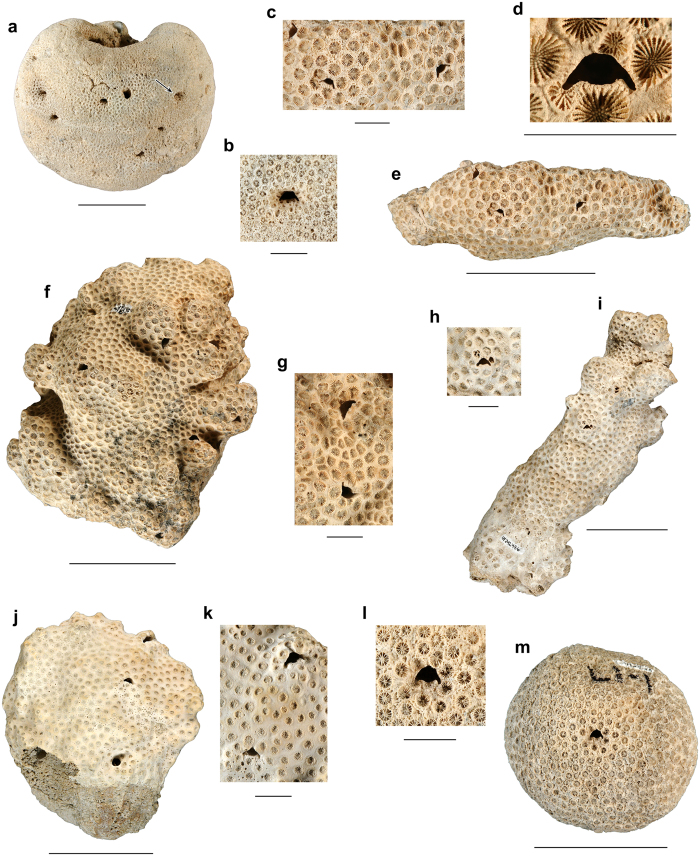
Specimens of *Solenastrea* spp. with crescentic pits. (**a**,**b**) Coral colony (UF 63964) and close-up of a pit (UF 257629) in *Solenastrea bournoni* from the lower-middle Pleistocene Caloosahatchee/Bermont Formations at Star Ranch 01, Palm Beach County, Florida. (**c**–**e**) Close-ups of three pits (UF 257624) and the coral colony (UF 242451) of *Solenastrea hyades* from the lower-middle Pleistocene Caloosahatchee/Bermont Formations at GKK Pit 01B, Palm Beach County, Florida. (**d**) = holotype *Galacticus duerri* igen. nov., isp. nov. (**f**,**g**) Coral colony (UF 242450) and close-ups of two pits (UF 257627) in *S. hyades* from the lower-middle Pleistocene Caloosahatchee/Bermont Formations at GKK Pit 01B, Palm Beach County, Florida. (**h**,**i**) Close-up of a pit (UF 257625) and the coral colony (UF 242456) of *S. hyades* from the lower-middle Pleistocene Caloosahatchee/Bermont Formations at Star Ranch 01, Palm Beach County, Florida. (**j**,**k**) Coral colony (UF 22265) and close-ups of two pits (UF 257621) in *S. hyades* from the upper Pliocene Pinecrest beds of the Tamiami Formation at Fiftymile Bend 01, Collier County, Florida. (**l**,**m**) Close-up of a pit (UF 257623) and the coral colony (UF 115115) of *S. hyades* from the middle Pleistocene Bermont Formation at Palm Beach Aggregates 02, Palm Beach County, Florida. Scale bar width = 50 mm for complete corals; 10 mm for close-ups.

**Figure 5:**
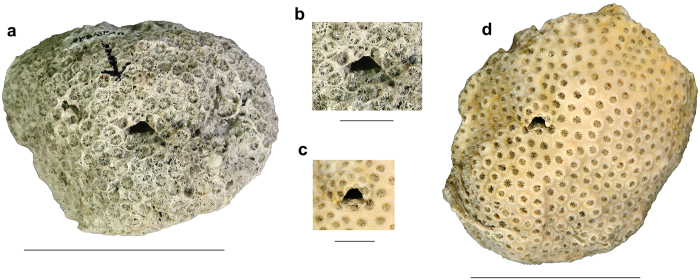
Specimens of *Solenastrea* sp. with crescentic pits. (**a**,**b**) Coral colony (UF 257525) and close-up of a pit (UF 257526) in *Solenastrea* sp. from the middle Pleistocene Bermont Formation at Palm Beach Aggregates 03, Palm Beach County, Florida. (**c**,**d**) Close-up of a pit (UF 257622) and the coral colony (UF 134135) of *Solenastrea* sp. from the upper Pleistocene Jaimanitas Formation at Caravela Road Fill Pit 01, Guantanamo Province, Cuba. Scale bar width = 50 mm for corals; 10 mm for close-ups.

**Figure 6:**
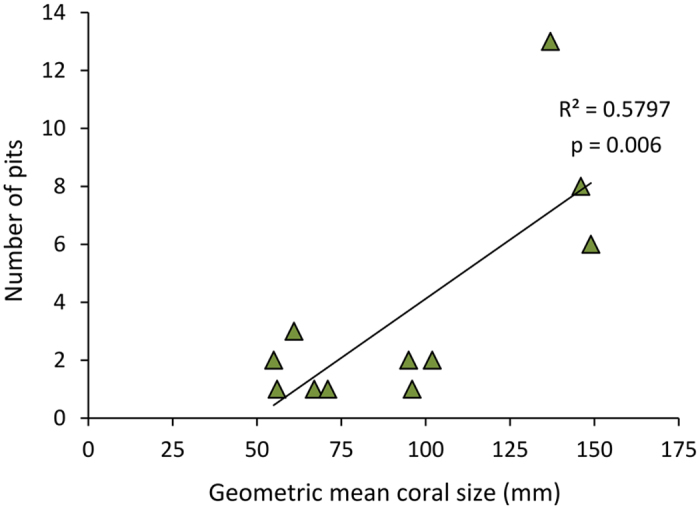
The geometric mean of the length (longest dimension here), width, and height of each coral specimen versus the number of domiciles. P-value calculated using a two-tailed t test (n = 11).

Ten of the fossil corals with crescentic pits originated from localities in southern Florida with nine collected from the lower–middle Pleistocene Caloosahatchee and Bermont Formations and the oldest one is found in the upper Pliocene Pinecrest beds of the Tamiami Formation. The 11^th^ specimen was collected from the upper Pleistocene Jaimanitas Formation of southern Cuba (Supplementary Figures S1, S2). In total, 11 coralla from two genera and five species (*Manicina areolata*, *Siderastrea* cf. *dalli*, *Siderastrea siderea*, *Solenastrea bournoni*, and *Solenastrea hyades*), together, yielded 40 crescent-shaped pits (Figs 2, 3, 4, 5, Supplementary Table S1). The corals ranged in size from 70 to 193 mm in longest dimension and consisted of various forms [e.g., massive heads, rotary (free-living), branching, and encrusting] (Figs 2, 3, 4, 5, Supplementary Table S1). The percentage of fossil corals with crescentic pits is rather low for specimens in the FLMNH collection (<0.1%), but targeted field work resulted in a higher percentage of such pits (2/~100 or ~2% of the specimens). The number of pits significantly increases with the size of the coral (Fig. 6), in agreement with results for *Cryptochirus coralliodytes*^41^, although their data is likely based on multiple cryptochirid species according to one of us (SETvdM).

### Description cryptochirid pits

On individual corals where multiple crescentic pits were located, none exhibited a strong preferred orientation (e.g., Figs 3,4). Many crescentic pits were hard to recognize when sediment-filled or when not viewed straight on. However, when cleaned of sediment, domiciles were unmistakably present. Furthermore, some corals were heavily bored by bivalves, polychaetes, sponges, etc., partially obscuring the crescentic pit (Fig. 3B). Pit interiors are lined (smooth with no coral structure visible, especially on the upper part) and unlined (coral structure visible), although lining could not be determined for all pits with certainty (Supplementary Table S1). Several pits appear to have thin septae at the rear of the chamber. The pits are oriented mostly parallel to the direction of coral growth without a depression around it, although most pits in some coral specimens have a depression around them (*Solenastrea bournoni*) or show the pit nearly perpendicular to the direction of coral growth (Figs 4J, 5D, Supplementary Table S1). No obvious relationship exists between the crescentic pit type^42^ and either the genus or coral type, although the crescentic pit type tends to be the same within most species. The lateral extensions of the pits are less distinct in fossil *Manicina areolata* specimens (Fig. 2). Pit shape can be affected somewhat by erosion (Fig. 5A).

**Figure 7:**
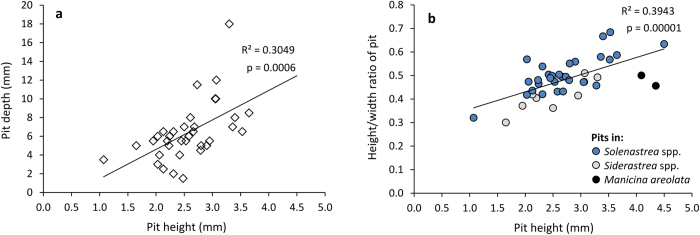
Relationships between pit height, depth, and the height/width ratio. (**a**) A significant positive relationship between pit height and depth. P-value calculated using a two-tailed t test. (n = 35). (**b**) Relationship between pit height and height/width ratio of the pit. P-value calculated using a two-tailed t test (n = 40).

The measurements of 40 pits range from 3.34–9.53 mm (mean = 5.65 mm) width and 1.07–4.35 mm height (mean = 2.74 mm) (Supplementary Table S1). Pit depth varies from 1.5–18.0 mm (mean = 6.39 mm), although the deepest pits could not be measured accurately. Pit diameter does not seem to narrow substantially towards the base. There is a positive significant relationship between pit height and depth (p = 0.0006, Fig. 7A), and also for pit width and depth (p = 0.0001), despite the fact that the precise depth of the deepest and largest pits in cross section could not be included. The height/width ratio significantly increases as width increases (p = 0.00001, Fig. 7B), a pattern not observed when height/width ratio is plotted against height (p = 0.99). A positive relationship between pit height and the height/width ratio exists also for subsets of the data: *Siderastrea* spp. (p = 0.01) and *Solenastrea* spp. (p = 0.000004).

### Shape of modern cryptochirid domiciles

A survey of images in the literature of modern shapes of cryptochirid domiciles shows that three basic forms can be identified: crescentic pits, circular to oval pits, and true galls or chambers (see Supplementary Table S1). We add the domicile shape of four species from which the pit shape was not yet reported (Supplementary Figure S3, Supplementary Table S1). (1) *Detocarcinus balssi* was found associated with three shallow crescentic pits in the lateral side of solitary coral *Anomocora marchadi* from the Eastern Atlantic, although it was suggested that pits of various shapes (circular, oval, and crescentic) in *A. marchadi* and *Caryophyllia smithii* from off the coast of Western Africa would have been produced by *D. balssi*^43^. Similar pits as in Supplementary Figure S3 were shown for the same coral species previously^44^. The other species include (2) *Dacryomaia japonica*, (3) *Lithoscaptus prionotus*, and (4) *Opecarcinus lobifrons* (Supplementary Figure S3). Each cryptochirid species produces only one of these three basic morphologies regardless of geographic location and the coral species they inhabit, suggesting that the crabs determine domicile morphology. Domicile shape is conserved within genera so that all species in relatively species-rich genera have a similar domicile shape (e.g., *Fizesereneia*, *Lithoscaptus*, *Opecarcinus*).

### Biogeography of modern cryptochirid domiciles

**Figure 8:**
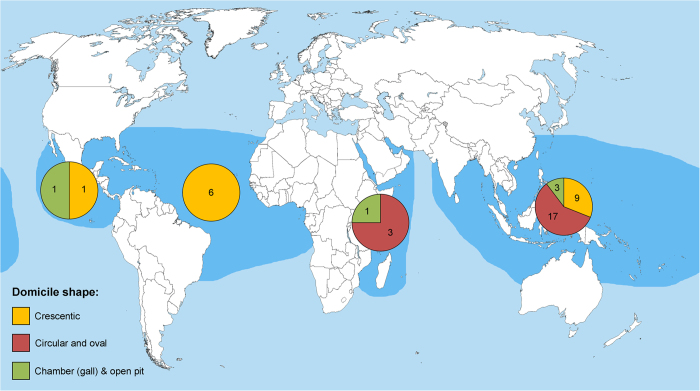
Geographic distribution of the three main domicile shapes of modern cryptochirid species and the number of species involved for each marine tropical province. Data in Supplementary Table S2. Tropical provinces follow Briggs & Bowen^85^,^86^,^87^. World base map from the Wikimedia Commons free media repository (https://commons.wikimedia.org/wiki/File:A_large_blank_world_map_with_oceans_marked_in_blue.PNG), released into the public domain by the authors, licensed under the Attribution-ShareAlike 3.0 Unported license (https://creativecommons.org/licenses/by-sa/3.0/), and modified in Photoshop 5, www.photoshop.com.

Extant cryptochirids inhabit domiciles in corals in tropical regions across the world, especially in the Indo-West Pacific (29/36 species or 81%, Supplementary Table S2). Crescentic domicile pits can be found in the Eastern Pacific (1 species), Atlantic (6 species), and Indo-West Pacific regions (9 species) (Fig. 8, Supplementary Table S2). Circular to oval pits are concentrated in the Indo-West Pacific region (17 species), whereas the two species that produce true galls (*H. marsupialis, P. ransoni*) are commonly found in all regions except the Atlantic Ocean.

## Discussion

### Likely culprits of fossil crescentic holes

Thus far two cryptochirid species were known from shallow marine habitats around Florida^18^: *Troglocarcinus corallicola* and *Opecarcinus hypostegus*. To this we add a third species: the FLMNH collections house two male and two female specimens of *Kroppcarcinus siderastreicola*, collected from *Siderastrea siderea*, from the Florida Keys (UF 24638–24641). These three crab species form crescentic cavities and have all been reported to inhabit *Siderastrea* in the Caribbean/Gulf of Mexico region (Mexico and Jamaica, resp.), whereas *T. corallicola* inhabits *Manicina areolata* in the same region in Belize, Colombia, Curaçao, Florida, Jamaica, and Mexico^18^,^35^,^45^,^46^,^47^,^48^,^49^. However, *K. siderastreicola* was only recorded from *Siderastrea*^9^,^35^. Thus, these crabs are considered to be the most likely culprits of the crescentic pits in at least part of the late Cenozoic corals from Florida and Cuba. The Pleistocene *M. areolata* is likely to have been inhabited by *T. corallicola*, and *K. siderastreicola* is the most likely candidate to have inhabited the fossil *Siderastrea* corals. *Solenastrea* has not been reported to be inhabited by any cryptochirids today^18^,^35^, implying that this species has not been found yet on modern *Solenastrea* or, perhaps less likely, does not live on this taxon anymore. To our knowledge, no cryptochirids have been reported from Cuba thus far, likely reflecting a lack of research, despite Cuba’s abundant reefs^50^. Pits are, however, figured in specimens of *Isophyllia sinuosa* and *Agaricia agaricites* from Cuba^51^. No mecha-nisms other than cryptochirid inhabitation are known to produce crescentic pits in corals. As such, these fossil pits are part of the ethological category *domichnia* (dwelling traces^52^,^53^).

### On cryptochirid growth and domicile shape and depth

Pit size correlates positively with size for extant cryptochirids, even when species from different genera are pooled^37^. Thus, pit size depicts specimen size. The increasing height/width ratio as pit size increases for the fossil domiciles studied (Fig. 7B) can be caused by gender differences for several reasons: (1) Oviparous females tend to be proportionally taller than males because their egg mass is carried under/around the abdomen (e.g., *O. hypostegus*^47^, *O. cathyae*^11^). (2) The chelae of various cryptochirids (e.g., *T. corallicola*, *O. hypostegus*, and *O. crescentus*) are proportionally larger and more robust in males compared to females^18^,^56^, implying that male specimens have a lower height/width ratio. (3) Female cryptochirids usually have a larger maximum size than males^18^,^46^,^54^,^55^, which also applies to cryptochirids known today from the Caribbean/Gulf of Mexico region^18^. Thus, the proportion of females should increase in larger size classes. While the first two reasons elucidate why crescentic pits of females have a greater height/width ratio compared to those of males, the third reason explains the trend of increasing height/width ratios as pit size increases.

Pit size significantly increases with pit depth (Fig. 7A), which is also observed based on a reanalyses of data from modern cryptochirid pits with a considerable size range (Supplementary Fig. S4). Except during mating activities^35^,^57^, the positive correlation may imply that cryptochirids stay in crescentic pits during their lifetime. Variability in the data could, at least in part, be explained by different species of corals that have different growth rates and variability in corallite growth on a single specimen. However, variation in coral growth cannot explain the increasing trend. Potts^36^ and Hiro^54^ argued against specimens inhabiting a single pit throughout their life for *Cryptochirus coralliodytes* because the specimens fit tightly in the pit and would molt and mate outside the pit, although this was not directly observed. This suggestion may be correct because a female of *T. corallicola* was figured partially outside of her pit with a male in close proximity^35^. Hiro^54^ mentioned that pits are uniform in size throughout, where an increase in size toward the entrance would be expected had the specimen lived near the entrance of the pit for a long time. Despite the risk of being preyed upon, the crab would settle on a living corallite or find a suitable pit to inhabit after molting according to Hiro^54^. However, Simon-Blecher & Achituv^41^ observed, in the lab, that a corallite dies after an adult crab settles on it. The pits herein do not narrow substantially towards the pit base, and the shallowest pits are not made by the smallest specimens herein (Supplementary Table S1), supporting Hiro’s hypothesis. Although the crabs usually sit at the entrance to close off the opening^36^,^56^, the uniform size allows the animal to withdraw as needed, for example when drying out^36^ or when predators approach. On one occasion, a cryptochirid was collected from the stomach of an apogonid fish^18^, but there are no further records of predation on cryptochirids. Hiro’s hypothesis could explain the correlations in Fig. 7A and Supplementary Figure S4, but only if cryptochirid growth slows as they mature. The crab would stay longer in each pit until the next molting phase, resulting in an increase in the mean pit depth for larger animals. A terminal anecdysis would also help to explain the pattern. The mode of cryptochirid growth (determinate, indeterminate) is not fully known^58^,^59^.

Another hypothesis can be proposed based on observations by Simon-Blecher & Achituv^41^. For *C. coralliodytes*, they documented that settlement into an existing pit is rather rare and was observed for young individuals (megalopa stage) only during a limited period, and sediments fill the pit when abandoned. Crucially, they also noted that the 30 female crabs never left their host in two years. Semper^60^ also observed the same species during a long period of its life and did not mention it changed pits or created a new one and one of us (SETvdM) never found a female cryptochirid outside her domicile. Simon-Blecher & Achituv^41^ speculated that spines on the first and second pairs of walking legs of *C. coralliodytes* can help to widen the pits as the crabs grow. Such spines are also present in other pit-forming cryptochirids, but not in the gall-producing *Hapalocarcinus marsupialis*^20^. Spines or tubercles are also absent from the carapace of *H. marsupialis*, but not in pit-forming cryptochirids, suggesting that this ornamentation may be functional in widening the pit as they grow. The other gall-forming cryptochirid, *Pseudohapalocarcinus ransoni*, exhibits sparse tubercles on the carapace and some spines on the appendages. These observations argue against Potts’ and Hiro’s hypothesis. Such spiny ornamentation of pit-forming cryptochirids could also help to widen the pit towards the base so that a more uniform size is obtained for withdrawal purposes. The spines and tubercles of cryptochirids differ in size and the sharpness of the spines and tubercles also vary. To what extent, if any, such ornamentation is able to abrade calcareous coral requires further investigation. This model of cryptochirids remaining in their pit throughout much of their life fits the positive correlations between pit size and depth (Fig. 7A, Supplementary Figure S4). More observations about pit depth, pit size throughout the pit, and crab behavior are needed for various species to test the two hypotheses.

The pits in *Detocarcinus balssi* are shallow (Supplementary Figure S3), as expected given the small size of the solitary coral and the relatively slow growth rate of the coral laterally. The positioning on the domiciles on the lateral side suggests that these crabs are not passive and do not wait for the coral to grow around them. Rather, *Detocarcinus balssi* likely uses a yet to be identified mechanism to create the pit. The crabs probably use a mechanism to lower the pH or produce an acid to dissolve the coral around them.

Domicile shape (crescentic, oval to circular, or true galls) may inform about male – female interactions. Crescentic and wide oval-shaped pits containing solitary females do not leave much room for a male. Either the female has to abandon the pit for mating (see above) or these cryptochirids are able to obtain sperm and store it for a long time prior to inhabiting a tight crescentic pit. Sperm longevity is unknown for cryptochirids, but varies from 3–48 months in other crabs^61^. More circular cavities leave opportunities for mating inside the pit [*Utinomiella dimorpha* (=*Pseudocryptochirus kahe*)^36^,^55^]. The latter authors mentioned that 28% of the females studied were accompanied by a male^55^. The galls of female *H. marsupialis* are still accessible for the free-living males in early (open) stages of gall formation, but not so once the gall is closed, leaving openings too small to enter for the male^62^. The crescentic fossil pits conform to the first scenario.

### Recognizing cryptochirid domiciles in the fossil record

To our knowledge, no other animal produces or induces crescentic pits in corals, implying that such pits can be confidently assigned to cryptochirids. Therefore, the specimens represent the first reported fossil traces of cryptochirids. These trace fossils are named *Galacticus duerri* igen. nov., isp. nov. (Supplementary Discussion S2). Some variation is observed in the coral morphology surrounding the pit. Nogueira *et al.*^42^ showed that a single cryptochirid species (*Kroppcarcinus siderastreicola*) on a single coral species (*Siderastrea stellata*) can create three domicile morphologies: (1) pit parallel to colony on a flattened surface; (2) pit parallel to colony, but surrounded by a depression; and (3) pit more or less perpendicular to the colony with an overhang. The morphology of all domiciles is still crescentic, underlining the conservative nature of domiciles within species^5^. Given the protruding nature of the overhang, this subtype has a lower preservation potential compared to the others.

Oval to circular pits in corals are not only produced by cryptochirids, but also by other invertebrates. For example, the thoracican barnacle *Lithotrya* can produce oval tunnels in reef rock^63^,^64^, circular perforations can be produced by the sponge *Siphonodictyon*^65^, and oval to circular to 8-shaped borings are bored by bivalves including *Lithophaga*^65^,^66^,^67^,^68^. Probable subcircular openings in corals can also be made by coralliophilid gastropods^66^,^69^, with the gastropod sitting directly underneath it in a larger cavity. Also other decapods can form oval to circular holes in corals: subcircular holes are produced by the shrimp *Pomatogebia operculata*^70^, by using their chelae^71^; oval to subcircular pits are made by the crab *Domecia acanthophora*^72^; subcircular holes are made the snapping shrimp *Alpheus*^73^; and elongate to subcircular holes are produced by the crab *Cymo*^74^. In conclusion, oval to circular pits in fossil corals cannot be used as evidence for the former presence of cryptochirids if not accompanied by body fossil evidence.

The development of galls by *Hapalocarcinus marsupialis* consists of several stages from open galls with a narrow opening to enclosed galls with several small openings^54^,^62^,^75^, and further variation is shown by others^76^,^77^,^78^,^79^. Considerable variation in gall shape is also observed in *Pseudohapalocarcinus ransoni*^80^,^81^. Galls are not exclusively made by cryptochirids, however. The crab *Tetralia* spp. was found to make gall-like structures in corals^74^, as does the shrimp *Paratypton siebenrocki*^82^. Galls of both decapods are found in Indo-West Pacific *Acropora*, a genus that is not reported to be inhabited by cryptochirids today. Copepods are also able to induce galls in stylasterid and pocilloporid corals^83^,^84^,^85^ that may resemble cryptochirid galls. All pocilloporid genera (*Pocillopora*, *Seriatopora*, and *Stylophora*) are also inhabited by *Hapalocarcinus marsupialis*^20^,^80^. In sum, the variation in shape of cryptochirid galls in conjunction with the often protruding nature of the gall, which makes the gall more prone to breakage and thus lowering its fossilization potential, and the fact that other crustaceans can produce galls makes it difficult to recognize them as traces of cryptochirids in fossil corals.

### Modern biogeography and evolution of cryptochirid domiciles

Crescentic pits by extant cryptochirids are found in the Atlantic, Eastern Pacific, and Indo-West Pacific (Fig. 8), but their fossil record is only partially known for the Western Atlantic based on results herein. Examination of Cenozoic fossil coral collections from all these regions should yield further examples of crescentic cryptochirid pits. This would help to constrain the antiquity of this cryptic crab family, specifically for members of genera that produce such pits: e.g., *Kroppcarcinus*, *Detocarcinus*, *Cecidocarcinus*, *Opecarcinus*, *Troglocarcinus*, *Pseudocryptochirus*, and to a lesser extent *Neotroglocarcinus* (Supplementary Table S2).

Wei *et al.*^37^ suggested that genera with crescentic pits are part of a clade within the Cryptochiridae, but their analysis was based on nine genera only, all collected near Taiwan. Van der Meij & Schubart^34^ used ten cryptochirid species in their phylogeny, but not all species producing crescentic pits cluster in a single clade, as was the case for a cladogram with 14 cryptochirid species^86^. Clearly, a more robust and complete phylogeny of cryptochirids is needed to test whether the habit of forming crescentic pits evolved once or multiple times.

### Cryptochirids in the fossil record

Recently, De Angeli & Ceccon^2^ reported on the first body fossils of a cryptochirid from the early Eocene (Ypresian) of Italy. However, Klompmaker & Boxshall^3^ pointed out several major differences between extant cryptochirids and *Montemagrechirus tethysianus*: (1) the rostrum of the Eocene species bears two spines, which is not seen in any extant species; (2) the orbits of *Montemagrechirus* are directed anterolaterally, which is highly unusual in extant species; and (3) the well-calcified nature of *Montemagrechirus* is remarkable in that symbiotic modern species receive protection from corals usually and often have a soft exoskeleton. Indeed, we did not find any cryptochirid remains in the domiciles herein. A difference not mentioned thus far is the presence of a faint cervical groove and associated pits axially in the Eocene species, features not clearly observed in extant cryptochirids. Additionally, the majority of modern cryptochirids have tubercles or spines on the carapace surface, whereas the De Angeli & Ceccon^2^ mentioned that the carapace surface is smooth. Thus, the high number of major differences between *Montemagrechirus* and modern cryptochirids suggest that placement of *Montemagrechirus* in the Cryptochiridae is not tenable. We here refer to it to a new family within the Brachyura: Montemagrechiridae fam. nov. (Supplementary Discussion S3). Consequently, we consider the late Cenozoic domiciles herein to be the oldest evidence of cryptochirids thus far.

## Conclusions

1. The first trace fossils of cryptochirids, 40 crescentic pits, are found in 11 Pleistocene and Pliocene corals from Florida and Cuba.
2. These pits may be the oldest evidence of cryptochirids in the fossil record; the claimed Eocene body fossils from Italy [*Montemagrechirus*] are deemed unconvincing and the genus is referred to a new family within the Brachyura: Montemagrechiridae fam. nov.
3. Pit size correlates significantly with pit depth, which may imply that the animals stay in their pit and widen it for a long time or that they move around while their growth slows down as they mature.
4. Pit height increases faster than pit width, which is probably related to the presence of a higher proportion of females in larger pits.
5. Coral size correlates significantly with the number of pits for those with pits.
6. Other cryptochirids domiciles (oval to circular and true gall) are likely difficult to recognize as trace fossils.
7. Modern biogeographic patterns of crescentic pits show that many more should be present in fossil corals from (sub)tropical regions around the world.
8. The crescentic trace fossils are named *Galacticus duerri* igen. nov., isp. nov.

## Additional Information

**How to cite this article**: Klompmaker, A. A. *et al.* Trace fossil evidence of coral-inhabiting crabs (Cryptochiridae) and its implications for growth and paleobiogeography. *Sci. Rep.* **6**, 23443; doi: 10.1038/srep23443 (2016).

## Supplementary Material

Supplementary Information

Supplementary Table S1

Supplementary Table S2

## Figures and Tables

**Figure 1:**
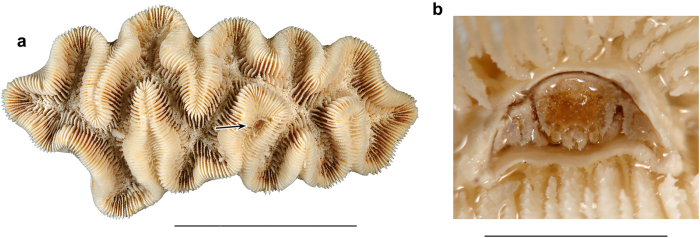
Modern cryptochirid crab in a crescentic pit in a coral. (**a**,**b**) *Troglocarcinus corallicola*, in the coral *Manicina areolata* (FSBC I 015641, largest coral specimen), locality unknown. Scale bar = 5.0 mm for (**a**); 50 mm for (**b**).
