# Supplementary material for: Trace fossil evidence of coral-inhabiting crabs (Cryptochiridae) and its implications for growth and paleobiogeography
Source: Sci Rep. 2016 Mar 24;6:23443. doi: 10.1038/srep23443 (PMC4806305; doi:10.1038/srep23443)
Supplement: Supplementary Information [file srep23443-s1.pdf]

Supplementary Information

Article in *Scientific Reports*

Trace fossil evidence of coral-inhabiting crabs (Cryptochiridae) and its implications for growth and paleobiogeography

Adiël A. Klompmaker, Roger W. Portell & Sancia E.T. van der Meij

Supplementary Discussion S1

Methods

The specimens reported herein are part of the Invertebrate Paleontology and Invertebrate Zoology Collections of the Florida Museum of Natural History (FLMNH) at the University of Florida (UF), Gainesville, Florida, USA. One modern coral specimen is deposited in the United States National Museum, Smithsonian Institution, Washington, D.C., USA (USNM), and one other specimen (Fig. 1) is from the Fish and Wildlife Research Institute, St. Petersburg, Florida (FSBC). After initial discovery of a fossil scleractinian coral exhibiting cryptochirid domiciles (UF 242451), an exhaustive search and examination of all well-preserved FLMNH Neogene and Quaternary corals (> 3,000 lots, > 9,000 specimens) from Florida, Cuba, and Jamaica was conducted. Additionally, field work at FLMNH-IP locality PB069 (Palm Beach Aggregates 03, Florida) was conducted to search for additional specimens. Approximately 100 corals from the Bermont Formation (middle Pleistocene) were examined at the locality and those with inferred cryptochirid domiciles were collected.

The domiciles were manually cleaned and checked for any crab remains. Subsequently, the maximum height along the midline, maximum width including the lateral extensions, and the depth of the pits were measured where possible. Various bivariate plots were made: (1) coral sizes as defined by the geometric mean of the length (longest dimension here), width, and height vs. number of domiciles (Fig. 6); (2) pit height or diameter vs. pit depth (Fig. 7A, Supplementary Fig. S4); and (3) pit height vs. height/width ratios of pits (Fig. 7B). A two-tailed t test was used to assess whether the ordinary least squares regression lines were significant. A p-value lower than 0.05 indicates that the trend is statistically significant. Furthermore, a literature research was conducted to determine the shape of the domicile of extant cryptochirid species, and an attempt was made to augment the dataset for species without domicile shape information by examining the collection of extant cryptochirids in the USNM (Washington, D.C.). Underwater pictures from recent fieldwork in the Indo-Pacific were added to the manuscript for cryptochirid species for which the domicile shapes were not yet recorded (Supplementary Figure S3).

Nomenclatural acts. This published work and the nomenclatural acts it contains have been registered in ZooBank, the proposed online registration system for the International Code of Zoological Nomenclature (ICZN). The ZooBank LSIDs (Life Science Identifiers) can be resolved and the associated information viewed through any standard web browser by appending the LSID to the prefix ‘http://zoobank.org/’. The LSIDs for this publication are urn:lsid:zoobank.org:pub:76A551C2-D094-43B6-BA1B-5EC179488DC7 (article), urn:lsid:zoobank.org:act:47F8036D-E54A-4762-9FDC-6C4DACDAF0D4

(Montemagrechiridae), urn:lsid:zoobank.org:act:CE15CE2B-AB65-4FD6-ACB2-F146FC11F9BC (*Galacticus*), and urn:lsid:zoobank.org:act:9B662C13-D4FA-454C-8262-C32F094863BD (*G. duerri*).

Supplementary Discussion S2

Systematic ichnology

Remarks: The crescentic pit is an example of bioclaustration, a process by which a living, skeletal-forming organism embeds in a living softer-bodied organism[1]. Bertling et al. (2006) cast doubt on whether bioclaustrations are trace fossils, but many authors disagreed and called such structures trace fossils and/or named them ichnotaxonomically subsequently[2–11]. Hybrid structures, resulting from boring and growth, have also been named ichnotaxonomically[12,13]. The cryptochirid crab *Cryptochirus coralliodytes* has been speculated to actively use their appendages to widen the pit[14], and many more species may do so given similar ornamentation on the appendages, including Western Atlantic species that produce crescentic pits[15,16]. We here treat the crescentic pits as trace fossils of cryptochirids.

Ichnogenus *Galacticus* nov.

Etymology: Derived from the battleship of Battlestar Galactica, an American science fiction franchise, because of the similar cross-sectional shape of this battleship to the pits. The gender is masculine.

Type ichnospecies: *Galacticus duerri* isp. nov.

Diagnosis: Cross-sectional shape of opening cavity crescentic in hard substrate.

Remarks: The ichnotaxobases as described in Pickerill[17], Bertling[18], and Tapanila & Ekdale[3] were used. We are unaware of trace fossils that have a similar crescentic shape[19–26], and, therefore, we erect the new ichnogenus and ichnospecies. A trace fossil with an elliptical to crescentic shape, *Osculichnus*, interpreted as a trace of a hunting fish in the sediment, consists of two lobes of different sizes[27]. Crescentic (meniscate), but repetitive, shapes can also be found as part of burrow backfills in soft substrates[28,29]. A semicircular shape may be produced by echinoids burrowing in soft substrates[21].

Galacticus duerri isp. nov. (Figs 2–5)

Etymology: Named after avocational paleontologist Richard Duerr (Okeechobee, Florida), who first found and donated a coral colony with trace fossils as recognized by RWP.

Type material and type locality: Holotype (Fig. 4D): Pit furthest away from other two of UF 257624 in *Solenastrea hyades* (UF 242451) from the lower-middle Pleistocene Caloosahatchee/Bermont Formations from the GKK Pit 01B, Palm Beach County, Florida, USA. Paratypes: Remainder of pits in close-ups of Figures 2–5.

Other material: Remainder of Supplementary Table S1 pits not figured in close-ups of Figures 2–5.

Diagnosis: Cross-sectional shape of cavity crescentic throughout single pit in calcareous substrate, wider than tall (height/width ratio ~0.3–0.7), central part lower margin straight to convex, lateral extensions narrow. Pit straight to slightly curved, does not widen or narrow toward deepest part (isodiametric). Cavity not penetrating substrate.

Description: Cross-sectional shape of cavity crescentic throughout single pit in calcareous substrate, wider than tall (height/width ratio ~0.3–0.7), central part lower margin straight to convex, lateral extensions narrow, often marking lowermost part when faced straight on. Pit straight to slightly curved, does not widen or narrow toward deepest part (isodiametric). Cavity not penetrating substrate, of variable depth, lined or unlined. Orientation relative to host mostly parallel to direction of growth of host.

Stratigraphic and geographic range: upper Pliocene – upper Pleistocene, Cuba and Florida.

Remarks: This trace fossil has only been found in corals thus far. Another host is not expected because modern cryptochirid crabs are only known to inhabit corals.

References

1. Palmer, T. J. & Wilson, M. A. Parasitism of Ordovician bryozoans and the origin of pseudoborings. *Palaeontology* **31**, 939–949 (1988).
2. Bertling, M. *et al*. Names for trace fossils: a uniform approach to concepts and procedures. *Lethaia* **39**, 265–286 (2006).
3. Tapanila, L. & Ekdale, A. A. Early history of symbiosis in living substrates: trace-fossil evidence from the marine record. in *Trace Fossils: Concepts, Problems, Prospects* (ed Miller III, W.) 345–355 (Elsevier, 2007).
4. Bromley, R. G., Beuck, L. & Ruggiero, E. T. Endolithic sponge versus terebratulid brachiopod, Pleistocene, Italy: accidental symbiosis, bioclaustration and deformity. in *Current Developments in Bioerosion* (eds Wisshak, M. & Tapanila, L.) 361–368 (Springer, 2008).
5. Tapanila, L. Direct evidence of ancient symbiosis using trace fossils. in *Paleontological Society Short Course 14: From Evolution to Geobiology: Research Questions Driving Paleontology at the Start of a New Century, Paleontological Society Short Course* (eds Kelley, P. H. & Bambach, R. K.) 271–287 (Paleontological Society, 2008).
6. McKinney, F. K. Bryozoan-hydroid symbiosis and a new ichnogenus, *Caupokeras*. *Ichnos* **16**, 193–201 (2009).
7. Knaust, D. Trace-fossil systematics. in *Developments in Sedimentology 64: Trace Fossils as Indicators of Sedimentary Environments* (eds Knaust, D. & Bromley, R. G.) 79–101 (Elsevier, 2012).
8. Santos, A. *et al*. Symbiotic association of a pyrgomatid barnacle with a coral from a volcanic middle Miocene shoreline (Porto Santo, Madeira Archipelago, Portugal). *Palaeontology* **55**, 173–182 (2012).

9. Cónsole-Gonella, C. & Marquillas, R. A. Bioclaustration trace fossils in epeiric shallow marine stromatolites: the Cretaceous-Palaeogene Yacoraite Formation, Northwestern Argentina. *Lethaia* **47**, 107–119 (2014).
10. Klompmaker, A. A., Artal, P., Van Bakel, B. W. M., Fraaije, R. H. B. & Jagt, J. W. M. Parasites in the fossil record: a Cretaceous fauna with isopod-infested decapod crustaceans, infestation patterns through time, and a new ichnotaxon. *PLOS ONE* **9**(3), e92551 (2014). doi:10.1371/journal.pone.0092551
11. Klompmaker, A. A. & Boxshall, G. A. Fossil crustaceans as parasites and hosts. *Adv. Parasit.* **90**, 233–289 (2015).
12. Vinn, O., Wilson, M. A., Mõtus, M. A. & Toom, U. The earliest bryozoan parasite: Middle Ordovician (Darriwilian) of Osmussaar Island, Estonia. *Palaeogeogr. Palaeoclimatol. Palaeoecol.* **414**, 129–132 (2014).
13. Wilson, M. A., Vinn, O. & Palmer, T. J. Bivalve borings, bioclaustrations and symbiosis in corals from the Upper Cretaceous (Cenomanian) of southern Israel. *Palaeogeogr. Palaeoclimatol. Palaeoecol.* **414**, 243–245 (2014).
14. Simon-Blecher, N. & Achituv, Y. Relationship between the coral pit crab *Cryptochirus coralliodytes* Heller and its host coral. *J. Exp. Mar. Biol. Ecol.* 215, 93–102 (1997).
15. Kropp, R. K. & Manning, R. B. The Atlantic gall crabs, family Cryptochiridae (Crustacea: Decapoda: Brachyura). *Smithsonian Contr. Zool.* **462**, 1–21 (1987).
16. Kropp, R. K. Revision of the genera of gall crabs (Crustacea: Cryptochiridae) occurring in the Pacific Ocean. *Pac. Sci.* **44**, 417–448 (1990).
17. Pickerill, R. K. Nomenclature and taxonomy of invertebrate trace fossils. in *The Palaeobiology of Trace Fossils* (ed. Donovan, S. K.) 3–42 (Johns Hopkins, 1994).
18. Bertling, M. What's in a name? Nomenclature, systematics, ichnotaxonomy. in *Trace Fossils: Concepts, Problems, Prospects* (ed Miller III, W.) 81–91 (Elsevier, 2007).
19. Crimes, T. P. & Harper, J. C. *Trace Fossils.* Geological Journal Special Issue 3 (Seel House Press, 1970).
20. Häntzschel, W. Trace fossils and problematica. in *Treatise on Invertebrate Paleontology, Part W* (eds Moore, R. C. & Teichert, C.) W1–W269 (Geological Society of America, University of Kansas, 1975).
21. Bromley, R. G. *Trace Fossils: Biology, Taxonomy and Applications, 2nd ed.* (Chapman & Hall, 1996).
22. Tapanila, L. Palaeoecology and diversity of endosymbionts in Palaeozoic marine invertebrates: Trace fossil evidence. *Lethaia* **38**, 89–99 (2005).
23. Hasiotis, S. T., Platt, B. F., Hembree, D. I., Everhart, M. J. & Miller, W. The trace-fossil record of vertebrates. in *Trace Fossils: Concepts, Problems, Prospects* (ed Miller, W. III) 196–218 (Elsevier Press, 2007).
24. Seilacher, A. *Trace Fossil Analysis* (Springer Science & Business Media, 2007).
25. Zonneveld, J. -P. & Gingras, M. K. *Sedilichnus*, *Oichnus*, *Fossichnus* and *Tremichnus*: 'Small round holes in shells' revisited. *J. Paleontol.* **88**, 895–905 (2014).
26. Wisshak, M. *et al*. In defence of an iconic ichnogenus – *Oichnus* Bromley, 1981. *Annales Societatis Geologorum Poloniae* (2015), doi: http://dx.doi.org/10.14241/asgp.2015.029

27. Demírcan, H. & Uchman, A. Kiss of death of a hunting fish: trace fossil *Osculichnus labialis* igen. et isp. nov. from late Eocene–early Oligocene prodelta sediments of the Mezardere Formation, Thrace Basin, NW Turkey. *Acta Geol. Pol.* **60**, 29–38 (2010).
28. D'Alessandro, A. & Bromley, R. G. Meniscate trace fossils and the *Muensteria-Taenidium* problem. *Palaeontology* **30**, 743–763 (1987).
29. Smith, J. J., Hasiotis, S. T., Kraus, M. J. & Woody, D. T. *Naktodemasis bowni*: new ichnogenus and ichnospecies for adhesive meniscate burrows (AMB), and paleoenvironmental implications, Paleogene Willwood Formation, Bighorn Basin, Wyoming. *J. Paleontol.* **82**, 267–278 (2008).

Supplementary Discussion S3

Systematic paleontology

Order Decapoda Latreille[1]
Infraorder Brachyura Linnaeus[2]
Superfamily uncertain
Family Montemagrechiridae nov.

Included genera: *Montemagrechirus* De Angeli & Ceccon[3]

Diagnosis: Well-calcified carapace longer than wide, narrowing posteriorly; large orbits directed anterolaterally; rostrum bearing two spines; carapace regions poorly defined, with faint cervical groove and pits axially; ornamentation weak to absent.

Remarks: Various major differences between *Montemagrechirus* and modern cryptochirids are observed (see above); hence, its placement in a new family. Superfamilial placement is more problematic because (1) few characters are available, (2) only the upper orbital margin is figured, (3) lateral sides are not figured, and (4) appendages and the ventral side are lacking. Some Raninoida show a similar overall carapace shape, but the frontal margin is usually more spinose, the upper orbital margins often contain notches, the lateral margin usually contains spines, and the rostrum does usually not contain two spines resembling *Montemagrechirus*. Various Corystoidea exhibit a bifid rostrum, but members of the superfamily differ on account of other reasons mentioned in the previous sentence. Although paguroid carapaces, especially the frontal, better calcified part, can exhibit a similar shape, the faint cervical groove in the middle of the carapace of *Montemagrechirus* suggests it is not a hermit crab. Moreover, paguroid rostra consist usually of a single spine and their orbits are directed forward. It cannot be entirely ruled out that the species is a precursor of the Cryptochiridae, and, if so, placement in the superfamily Cryptochiroidea would be best. However, more well-preserved specimens and other species would be needed.

References:

1. Latreille, P. A. *Histoire naturelle, générale et particulière des crustacés et des insects, vol. 3* (F. Dufart, 1802–1803).

2. Linnaeus C. *Systema naturae per regna tria naturae, secundum classes, ordines, genera, species, cum characteribus, differentiis, synonymis locis, vol. 1. Ed. 10* (Laurentius Salvius, 1758).
3. De Angeli, A. & Ceccon, L. Nuovi crostacei brachiuri dell'Eocene di Monte Magrè. (Vicenza, Italia settentrionale). *Lavori Società Veneziana di Scienze Naturali* **40**, 119–138 (2015).

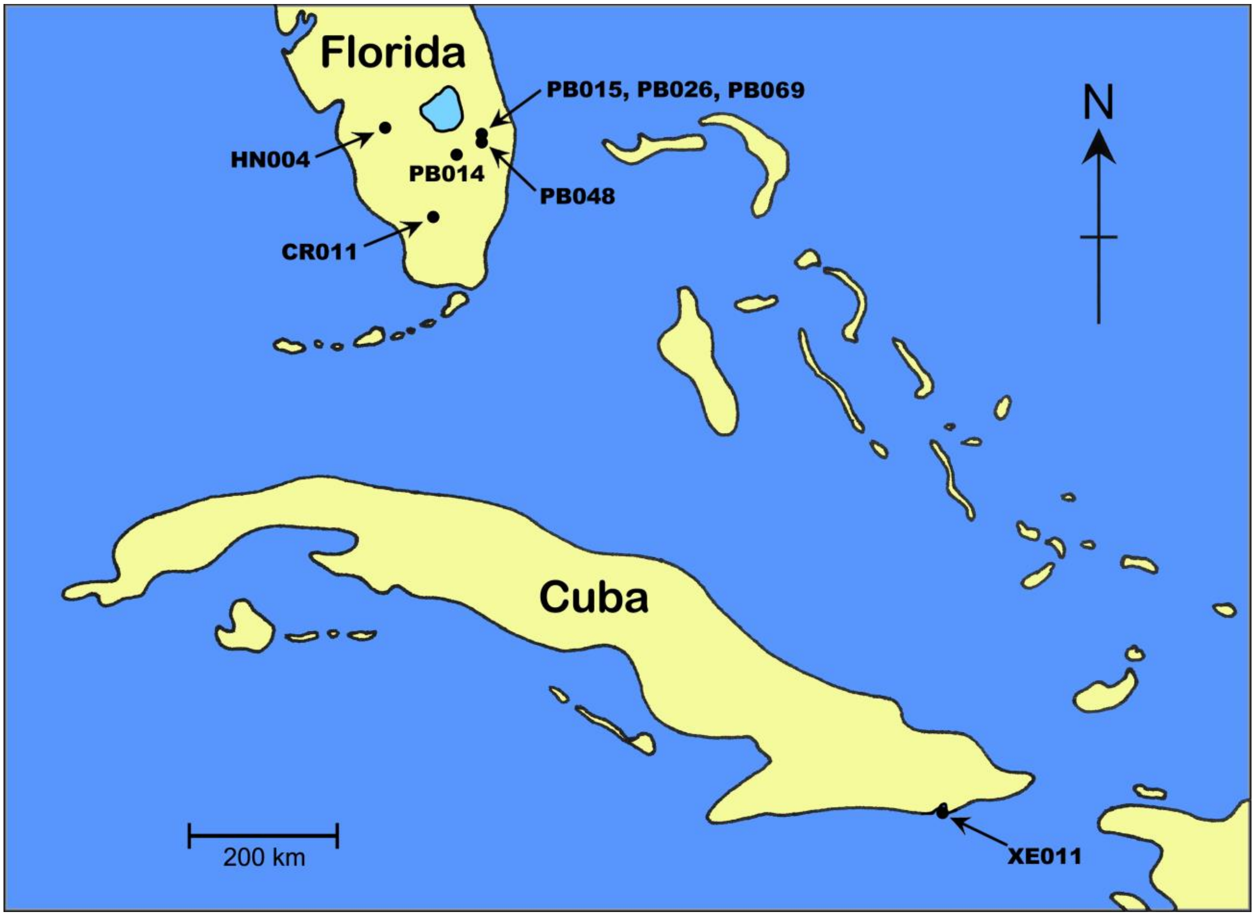

Supplementary Figure S1. Map with fossil cryptochirid pit localities in Florida and Cuba. FLMNH-IP site keys: HN004 = Cochran Shell Pit, Hendry County, Florida; PB015 = GKK Pit 01, Palm Beach County, Florida; PB026 = GKK Pit 01B, Palm Beach County, Florida; PB069 = Palm Beach Aggregates 03, Palm Beach County, Florida; PB048 = Palm Beach Aggregates 02, Palm Beach County, Florida; PB014 = Star Ranch 01, Palm Beach County, Florida; CR011 = Fiftymile Bend 01, Collier County, Florida; XE011 = Caravela Road Fill Pit 01, Guantanamo Province, Cuba. Map was hand drawn in Photoshop 6, www.photoshop.com.

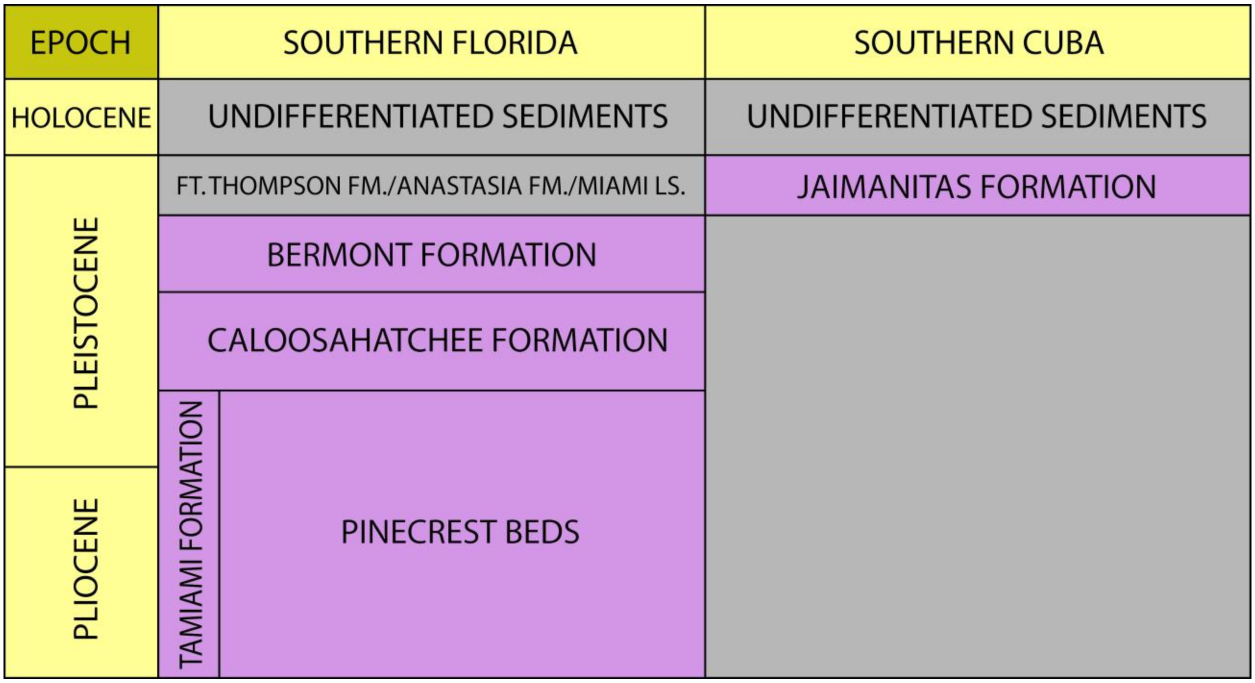

Supplementary Figure S2. Stratigraphic column of southern Florida and Cuba. Units with corals containing cryptochirid pits in purple. The Pinecrest beds of the Tamiami Formation at the locality CR011 are late Pliocene in age.

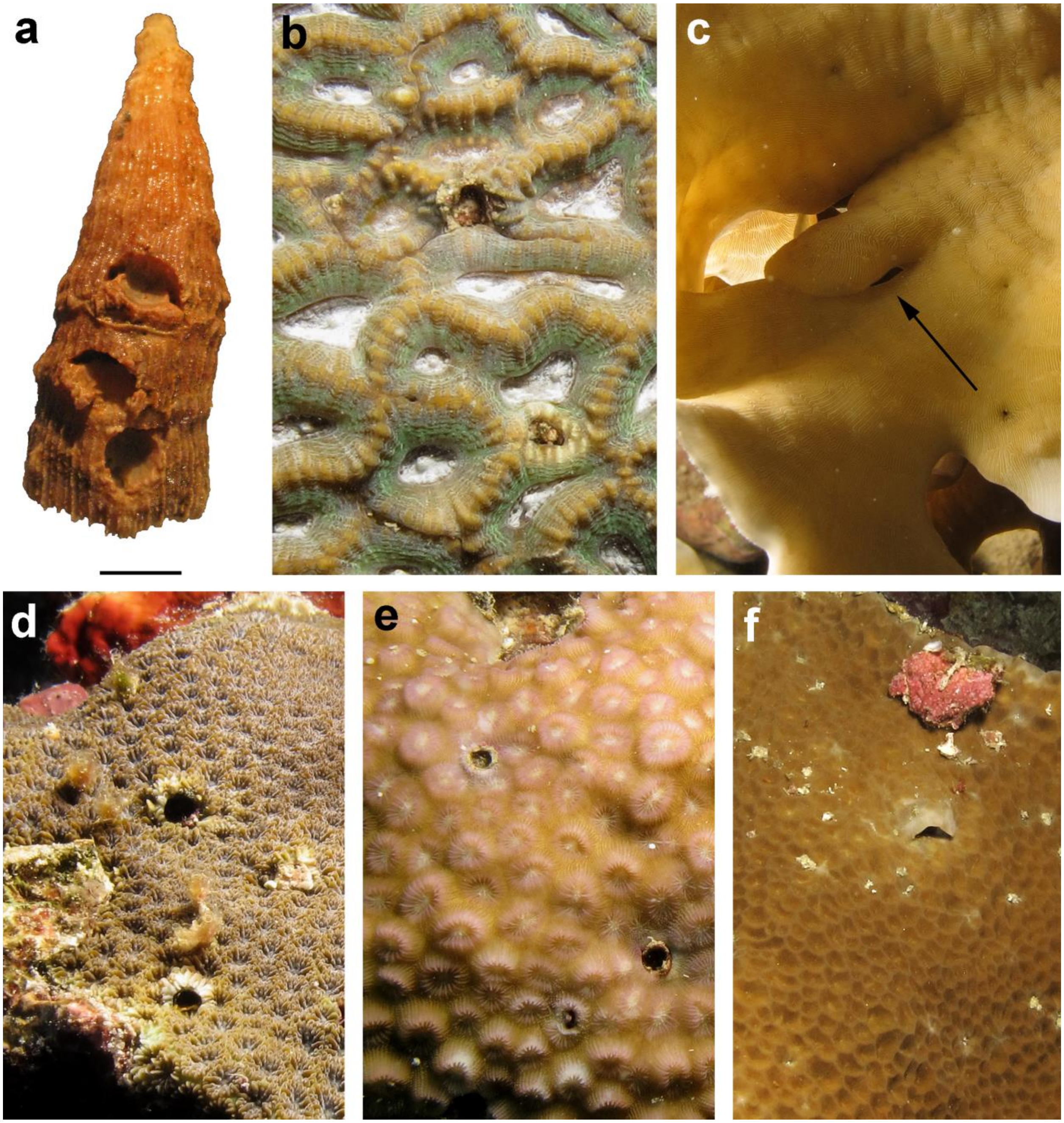

Supplementary Figure S3. Domiciles shapes of various modern cryptochirid taxa embedded in corals. (**a**) Solitary coral of *Anomocora marchadi* (USNM 234253), dredged from 50–55 m depth in the Eastern Atlantic (Gulf of Guinea), with three crescentic pits associated with the cryptochirid *Detocarcinus balssi*. Coral is shown upside down. Scale bar width = 5.0 mm. (**b**) *Oulophyllia bennettae* from the Tun Mustapha Park, Northern Borneo, Malaysia, with two circular to oval pits of *Lithoscaptus prionotus.* (**c**) *Pavona cactus* from the Tun Mustapha Park, Northern Borneo, Malaysia, with a cryptic gall (see arrow) of *Pseudohapalocacinus ransoni*. (**d**) *Leptastrea* sp. from the Faafu Atoll of the Maldives with two circular to oval pits of *Dacryomaia japonica*. (**e**) *Astrea curta* from the Tun Mustapha Park, Northern Borneo, Malaysia, with three circular to oval pits of *Sphenomaia pyriformis.* (**f**) *Gardineroseris planulata* from the Faafu Atoll of the Maldives with a crescentic pit of *Opecarcinus lobifrons*.

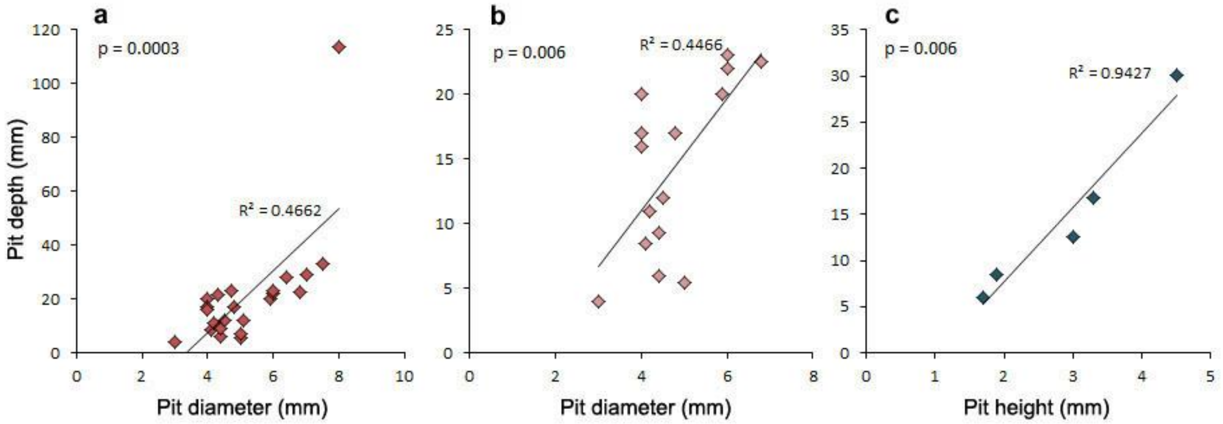

Supplementary Figure S4. Positive significant correlations between cryptochirid pit size and pit depth using data from Hiro (1937: table 4) and Garth & Hopkins (1968, p. 42). (**a**) Pits of "*Cryptochirus coralliodytes*" (likely several species) found in various corals, n = 23. (**b**) Subset of (**a**) with pits of "*C. coralliodytes*" in *Platygyra lamellina*, n = 15. (**c**) Pits of *Opecarcinus crescentus* in *Pavona gigantea*, n = 5. P-values calculated using two-tailed t tests.

Supplementary Table S1. Data on fossil corals with crescentic cryptochirid pits. (see Excel file)

Supplementary Table S2. Data on the shape of the domiciles of modern cryptochirids. (see Excel file)
